# A comparative study of national variations of the European WEEE directive: manufacturer’s view

**DOI:** 10.1007/s11356-021-13206-z

**Published:** 2021-03-05

**Authors:** Terje Andersen

**Affiliations:** Molde University College, Specialized University in Logistics, P.O. Box 2110, NO-6402 Molde, Norway

**Keywords:** Waste electrical and electronic equipment (WEEE), E-waste, Reverse supply chain, Manufacturer, Circularity, Producer responsibility organization (PRO), Circular economy (CE), Case study

## Abstract

We are facing the challenge of rapid growth in waste from electrical products (e-waste). In Europe, handling e-waste is regulated by the European Waste Electrical and Electronic Equipment (WEEE) directive, which is based on the extended producer responsibility (EPR) model as a regulatory tool forcing manufacturers and importers to take responsibility for their products throughout their lifecycles. However, the directive allows for great variations in implementations in each country, causing e-manufacturers and e-waste handling operators to face challenges in their transition to more sustainable operations. To identify the challenges involved, this study investigates the effect of the WEEE directive from a manufacturer’s perspective. A case study of an e-manufacturer operating subsidiaries in several European countries and the associated producer responsibility organizations (PROs) is presented. The case study includes interviews from 17 stakeholders in 12 organizations in eight European countries. Key findings are as follows. First, the WEEE data reported are not harmonized. Second, the calculations of the environmental fee differ across countries. Third, following up on different national WEEE obligations sometimes leads to over-reporting to avoid negative effects on environmental corporate social responsibility, brand reputation, and profitability. Fourth, outsourcing end-of-life (EoL) treatment responsibility to PROs is seen as positive by the manufacturer but results in a decoupling of the EPR and the operational EoL treatment, which may reduce efforts to transfer to a higher circularity level of its EEE products. Fifth, WEEE is considered a way for e-manufacturers to handle waste not to adopt a circular focus. This paper contributes to both practitioners and researchers within reverse logistics and sustainability by adding knowledge from real-life context of how EPR is implemented in WEEE.

## Introduction

There is an increased focus on achieving a more sustainable future. Sustainability entails not only environmental protection but also economic and social dimensions (Brundtland et al. 1987), known as the triple bottom line (Elkington 1998). One method to achieve sustainability is to move from a traditional linear economic model characterized by a make-use-dispose approach to a circular economy (CE) model, in which materials and energy remain in a restorative system (Geissdoerfer et al. 2017). CE is the transition moving from a linear economic approach to a circular economic model where materials and energy flow back into the economy after end-of-life (EoL) with ideally zero waste. Technical material should flow in circles so they can be maintained, re-used, refurbished, or recycled, leaving waste to be eliminated or at least minimized (Ellen-MacArthur-Foundation, (EMF) 2013). This process is often visualized as a loop, a perfect environmental regenerative circle. To reach sustainability goals, e-waste is of particular interest. E-waste is among the world’s fastest-growing waste streams, fueled by higher consumption rates of e-equipment, short life cycles, and limited repair rates, as reported by the UN Global E-waste Monitor 2020 (Forti et al. 2020). Increasing levels of e-waste pose significant challenges to the achievement of the UN’s sustainability goals (Lee et al. 2017; Forti et al. 2020). In Europe, handling e-waste is regulated by the Waste Electrical and Electronic Equipment (WEEE) directive (Union 2012). This directive is based on the producer responsibility model as a regulatory tool to force manufacturers and importers to take responsibility for their products over their entire lifecycles. However, the directive allows for significant variations in implementation among countries, resulting in each country having its own national rules. For e-manufacturers typically operating in many countries, the variations in national implementations of the same WEEE directive implies additional management efforts and increased costs, slowing the progress toward the sustainability goals.

Islam and Huda (2018) performed a literature review on WEEE from a logistics perspective, focusing on reverse logistics and closed-loop supply chain (CLSC), and identified a set of research gaps including a lack of empirical research on real-world cases and qualitative research providing an in-depth understanding of practical problems and specifying policy implications for authorities. Andersen et al. (2020) studied Norwegian and Danish e-manufacturers’ approach to circularity in the WEEE directive and found a need to focus on national variations in WEEE implementation. Bressanelli et al. (2020) conducted a systematic literature review of 115 papers on the CE in the WEEE industry and identified research gaps that call for attention to five aspects:

1. Research has been mainly explorative, targeting impact evaluation by authorities and leading to a call for research studies that “demonstrate how CE can be applied to the WEEE industry to solve practical problems, through empirical theory-testing and validation research.”
2. Research using quantitative approaches for assessment dominates, and no research in the WEEE industry has focused on collecting data from a large set of companies, leading to a need to “investigate CE in the WEEE industry by combining quantitative and qualitative approaches (such as survey and case studies) focused on companies and supply chains.”
3. Top-down approaches to sustainability are dominant even when companies are willing to implement a CE strategy without regulatory rules if it generates economic, social, and environmental advantages. This finding calls for research studies that “investigate, quantify and validate the mechanisms for how CE changes companies’ behavior from top-down to bottom-up.”
4. There is a lack of research on retailers and service providers, leading to a need for studies that “explore the role and the CE implications for retailers and service providers in the WEEE industry.”
5. Overall, research lacks joint consideration of several supply chain actors and lifecycle phases leading to a call to “consider all WEEE ecosystem’s actors and all life cycle phases simultaneously.”

Doan et al. (2019) focused on e-waste reverse supply chains in their review of papers in the four main groups: factors of implementation, performance evaluation and decision-making, fostering product returns, and network design. Of particular interest for manufacturers is the factors of implementation group where they identified a gap in how the extended producer responsibility (EPR) policy affects e-manufacturers’ EoL treatment of their products. They call for more practical research on e-waste implementations with an industry perspective linked to implementation at the national level.

To summarize, there is a lack of empirical research on real-world cases that include the effect of national variations in WEEE implementation with a focus on companies and service providers. This paper addresses this gap through the following research problem:

*How are e-manufacturers affected by national variations in the WEEE directive implementation?*

### National implementations of the WEEE directive and producer responsibility organizations (PROs)

European e-manufacturers need to follow the WEEE directive that entered into force on 13 August 2012. The 2012 directive required each country to transpose it into national law by 14 February 2014, leading to the same directive being effectuated in different ways in each country. A transitional period was defined between 13 August 2012 and 14 August 2018 to include all requirements.

Within the European Union (EU) legislations covering e-waste management, there are two overall principles. One, known as extended producer responsibility (EPR), is that producers need to take responsibility for the EoL phase of the products they make (OECD 2017). The other, known as the polluter pays principle (PPP), states that the one responsible for the waste has to pay the cost of handling the waste in a decent way.

The WEEE directive is based on the EPR and PPP principles. However, since the EPR principle allows manufacturers to delegate the operational EoL treatment to a third party, a new type of organization has emerged: a producer responsibility organization (PRO) (Fleckinger and Glachant 2010; Wang et al. 2017). How a PRO is organized varies depending on country since the national translation of the WEEE directive varies for each country.

To investigate the main research problem, the PROs were included in the study. To guide this research, five research questions (RQs) were formed. In the RQs, both the e-manufacturer and the PRO are denoted as e-actors:

1. How does a national e-actor view its e-waste handling related to the WEEE regime?
2. How has a national e-actor reacted to changes in the WEEE directive?
3. How does a national e-actor respond to the increased circularity focus in the WEEE directive?
4. How is the WEEE directive managed from a national e-actor’s perspective?
5. How do national variations in the WEEE directive implementation affect e-actors?

Answering these research questions will contribute to both practitioners and researchers within reverse logistics and sustainability. On an operations/management level, the study will provide industry practitioners with insight into how other industries handle their EPR obligations. This will be valid both for industries working under the European WEEE legislation, industries working under other EoL legislations, and industries not regulated by EPR legislations. For policy-makers, the study provides insight into how an international manufacturer reacts to EoL legislation and gives advice based on the findings. From a research perspective, the study will contribute to knowledge from practical examples of how EPR is implemented in different industries.

The paper is structured as follows. In Section 2, the literature overview and background information on e-waste handling according to the WEEE regime, the circularity of EEE products, and the EPR are presented. In Section 3, the research method is explained, followed by a description of the case company and the PROs in Section 4. The study’s results are presented in Section 5. Section 6 analyzes and discusses the results before conclusions, implications, limitations, and directions for future research are presented in Section 7.

## Literature overview and background

E-waste has received attention from the scientific community. A recent bibliometric analysis performed by Zhang et al. (2019) identified 2847 publications, most in recent years, within the area. These publications are spread over 627 journals. Although the number of journals is large, the top 10 journals have over 40% of the publications. Most of the publications are within the field of environmental science. Cesaro et al. (2018) remark on the historical research focus on metal recycling and recovery. To improve WEEE management, they support for interdisciplinary research along with transnational approach. Zlamparet et al. (2017) argue that the majority of the e-waste studies focus on recycling, which is rated as the least sustainable option according to the CE model (Ellen-MacArthur-Foundation 2013). Several literature reviews about the phenomenon have been conducted, among them Pérez-Belis et al. (2014), Raja and Gandhi (2014), Long et al. (2016), and Andrade et al. (2019). These studies focus on WEEE from different perspectives.

European manufacturers and importers of EEE have been covered by the European WEEE directives (Union 2002, 2012) for nearly two decades. The first directive, from 2002, was implemented in the European countries during that decade. The original deadline to implement the directive into national laws was 2004, but several member states were not able to meet this date (Savage et al. 2006). Other European countries established compliance schemes before this time (Ylä-Mella et al. 2014). The updated directive with a deadline for implementation on national laws set to 14 February 2014 also had some delays. The original WEEE directive (Union 2002) was closed scope, only covering specific product categories. Products outside these categories were not part of the legislation. The updated directive (Union 2012) is an open scope directive. All WEEE, except those specifically mentioned, are covered by the legislation. There was a long transition period, from 2012 to 2018, for this transmission. This included a change in the classification of EEE, from the original ten closed product categories introduced in 2002 to the six new open product categories valid from 15 August 2018. The new directive also set higher targets when introducing a new target deadline of 15 August 2015 and with higher targets on the new categories valid from 15 August 2018. In addition, the target “recycled,” used until 14 August 2015, was changed to “prepared for re-use or recycled,” indicating an increased focus on circularity in EEE. Although the introduction of the new re-use term was introduced in 2015, there are no specific re-use targets in the directive (González et al. 2017). However, each EU country may implement more ambitious national targets. Only Spain has presently implemented specific re-use targets (McMahon et al. 2019). Table 1 provides the historical development of recover, recycle, and prepare for re-use or recycle targets within the WEEE directive. Table 2 presents the old and current product categories within the directive. A more detailed list of the categories (old and current) and their contents are listed in the appendix. There is also an annual minimum collection rate in the directive. Until 2018, the collection rate needed to be at least 45% of the EEE on the market. This figure increased to 65% as of 2019. Eurostat figures for 2017 indicate that most European countries performed according to the directive, at least on an overall level (Eurostat 2017).

**Table 1:** Historical WEEE targets (adapted from Union 2012 and Islam and Huda 2018)

10 closed categories before 15 August 2018					6 open categories after 15 August 2018		
	Targets before 15 August 2015		Targets after 15 August 2015			Targets after 15 August 2018	
Category	Recovered (%)	Recycled (%)	Recovered (%)	Prepared for re-use or recycled (%)	Category	Recovered (%)	Prepared for re-use or recycled (%)
1	80	75	80	80	1	85	80
2	70	50	75	55	2	80	70
3	75	65	80	70	3	–	80
4	75	65	80	70	4	85	80
5	70	50	75	55	5	75	55
6	70	50	75	55	6	75	55
7	70	50	75	55			
8	70	50	75	55			
9	70	50	75	55			
10	80	75	85	80

**Table 2:** WEEE categories (adapted from Union 2012 and Islam and Huda 2018)

WEEE categories before 15 August 2018	WEEE categories after 15 August 2018
1 Large household appliances	1 Temperature exchange equipment
2 Small household appliances	2 Screens, monitors, equipment with surface screens > 100 cm^2^
3 IT and telecommunications equipment	3 Lamps
4 Electronic and consumer equipment	4 Large equipment (any external dimension more than 50 cm)
5 Lighting equipment	5 Small equipment (no external dimension more than 50 cm)
6 Electrical and electronic tools	6 Small IT and telecommunications equipment (no external dimension more than 50 cm)
7 Toys, leisure, and sports equipment	
8 Medical devices	
9 Monitoring and control instruments	
10 Automatic dispensers	

**Fig. 1:**
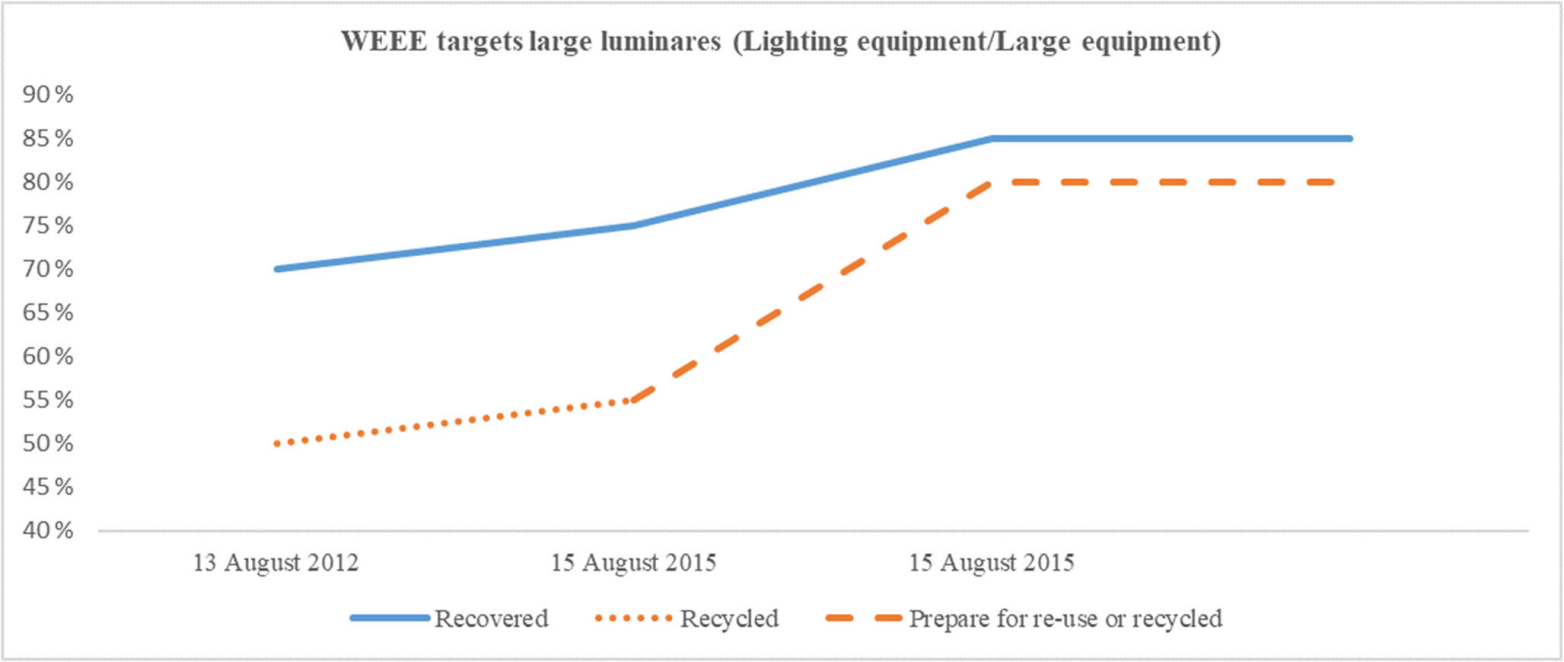
Historical recovery and recycled targets, lighting equipment

**Fig. 2:**
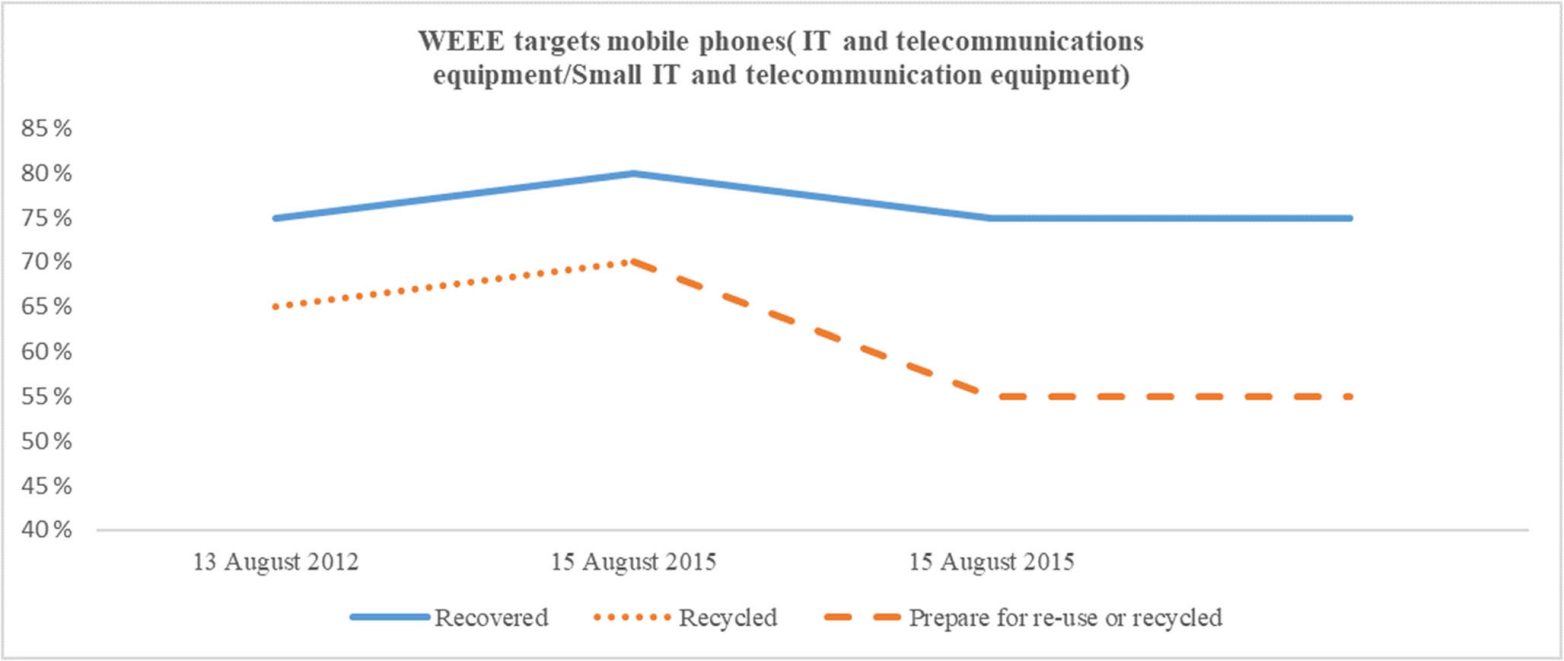
Historical recovery and recycled targets, mobile phones

As illustrated in Table 1, the target changes over the years are all either higher targets or status quo. The recovery targets started on levels between 70 and 80% and increased to 75 to 85% in 2015, remaining in this range. The recycled/prepared for reuse targets started at 50 to 75% before 2015, increased to 55 to 80% in 2015, and today, three of six product categories have 80% targets. However, the reclassification in the WEEE directive makes the actual new collection targets *lower* than the old ones for some products. Thus, investigating the historical development of the targets is challenging. Products that fit one specific old category may move to different new categories based on the products’ sizes. In Figs. 1 and 2, the historical development targets are visualized for two products. Figure 1 depicts the development for a large lighting luminaire (> ⌀ 50 cm), and Fig. 2 depicts the development of a mobile phone. As illustrated, the reclassification does not always result in increased targets. The large lighting luminaire from the old category 5, lighting equipment, moved to the new category 4, large equipment, resulting in an improved target rate of recovery and prepare for re-use or recycled.

Figure 2 focuses on the reclassification of mobile phones, which were originally classified as IT and telecommunications equipment (category 3) with targets 75%/65% on recovered/recycled before 2015 and 80%/70% in 2015. With the new category (small IT and telecommunications equipment), the targets dropped to 75%/55%. Figures 1 and 2 illustrate that despite the recovered and recycled targets normally increasing over time, the new categorization of WEEE products may have the opposite effect. The changes, including the recycle target from “recycled” to “prepare for re-use or recycled,” make the historical target development challenging to compare.

When reporting according to the WEEE targets, the basic recovery rate calculation is recovery rate equals the weight of EoL EE-products collected divided by the weight of new EE-products placed on the market. This metric is a rough estimate since the total number of products on the market contributes to the number collected, while the number of new products placed on the market is calculated per year. Manufacturers and importers collect a fee when selling EEE equipment. The fee may be called an environmental fee, EoL fee, recycle fee, and so on (Ongondo et al. 2011). This fee finances the collection and treatment of the waste, which normally is completed by PROs. These PROs can be organized as either several competing collective systems or as one non-competing collective system (Corsini et al. 2017; Dieste et al. 2017).

As opposed to supply chain management, reverse supply chain management includes the activities required to return a product from a customer and either dispose of it or input it in some form or reuse or recovery process (Guide Jr. and Van Wassenhove 2002; Prahinski and Kocabasoglu 2006). This forward and reverse supply chain is a loop if there is a relationship between markets for the returned product (Salema et al. 2007). The loop is closed if the two markets coincide and is open if they do not. The difference is also illustrated by the actors in the loop. In an open-loop supply chain, the actors placing products on the market are not the same as those retrieving the products after EoL for reuse/recovery; in a closed loop, the actors are the same (Gou et al. 2008).

**Fig. 3:**
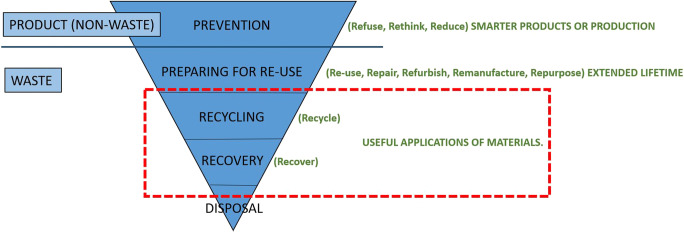
EU’s waste hierarchy (blue), circular strategies (green), and the EU WEEE directive (red). Adapted from Potting et al. (2017) and Union (2008b, 2012)

The EU waste directive aims to prevent waste. Preparing for re-use is the most preferred waste option, followed by recycling and recovery. Disposal at landfill is the least preferred option for an EoL product (Union 2008b). Likewise, CE’s goal is to eliminate waste (Ellen-MacArthur-Foundation, (EMF) 2013; Union 2015), but here, the higher levels in the hierarchy have potential for economic growth. Within waste handling, the 4R approach (reduce, recycle, recover, and reuse) is well known. Potting et al. (2017) expanded this approach to a 10R approach (recover, recycle, repurpose, remanufacture, refurbish, repair, re-use, reduce, rethink, refuse), identifying ten CE strategies in which disposal is not an option, one clear goal of CE. They divide the strategies into three overarching levels: (1) smarter products or production, (2) extended lifetime of products, and (3) useful applications of materials. The WEEE directive focuses on the waste part, the less preferred option in both the waste hierarchy and CE strategies. A combination of the different CE strategies, WEEE treatment according to the WEEE directive, and the EU waste hierarchy is presented in Fig. 3. The blue portion represents the waste hierarchy, the green is the circular strategies, and the red is the WEEE directive. As illustrated in Fig. 3, the WEEE directive focuses on the lower sections of the waste hierarchy and circular strategies. Other directives focus more on CE, among them the European Eco design directive (Union 2008a).

## Research methodology

To investigate how e-manufacturers are affected by national variations in the WEEE directive, an e-manufacturer with subsidiaries in several European countries was selected. The e-manufacturer is affected by the individual national implementation of the WEEE directive. To investigate national variations, we focused on one e-manufacturer, in one industry, with one type of products. Many of the characteristics are the same, but the implementation of the directive and how the organization of PROs is achieved vary.

To answer RQ1 through RQ5, a case study was conducted (Yin 2018). This study consisted of semi-structured interviews with employees at each of the e-manufacturer subsidiaries, as well as employees at the associated PROs. However, some of the PROs were not able or willing to participate in the research.

The interviewed stakeholders were from different areas within the e-manufacturer’s organization, among them accounting (A), controlling (C), quality management (QM), product approval (PA), product development (PD), product management (PM), and sales (S).

The stakeholders represented the management within the different streams, with both national and international stream responsibilities within the case company. All stakeholders had full decision-making rights within their part of the WEEE legislation, but due to differing organizations of the WEEE treatment, the stakeholders were from different streams. Although the WEEE directive applies to all countries under the regulation, directive handling within each country might vary. To investigate this possible variance, seven countries (Norway, Denmark, Germany, the UK, Ireland, the Netherlands, and Estonia) with subsidiaries of a global EEE manufacturer were selected. Using this wide European approach enabled the identification of the national variations of the implementation of the WEEE directive and how an EEE manufacturer responds to its WEEE obligations. Using one EEE manufacturer, with operations in different European countries, allowed the study to focus on a manufacturer’s view of the national variations of the WEEE directive.

Since the case company’s head office is located in Norway, the panel selection started with an in-person interview with the manager responsible for WEEE reporting in Norway. She included one of her key employees within WEEE who did all the reporting. She also included the Health, Environmental, and Safety/Quality Manager in the interview. These three employees could answer many of the interview questions. Product development and product approval is a centralized function for the company. Questions related to these topics were answered during a follow-up interview with stakeholders within these areas. These interviews were conducted over the phone. Each national subsidiary in the case company was responsible for fulfillment of its national WEEE obligations. Based on this information, subsidiaries in Denmark, Germany, the UK, Ireland, the Netherlands, and Estonia were also interviewed. However, many of the answers received from Norway related to product development and declarations of products, and certificates were also valid in these countries. The stakeholders in the rest of the countries were partly identified during the interviews in Norway and partly in dialogue with the subsidiaries in the other six European countries. Parts of the interviews covering Norway and Denmark were part of another study conducted by the same author (Andersen et al. 2020) in February, March, and May 2020. Follow-up interviews in these countries, and new interviews in the rest of the countries, were concluded between June 2020 and October 2020. Due to the COVID-19 pandemic, physical interviews were put on hold. All interviews completed after March 2020 were phone interviews. All interviews were recorded, transcribed, and sent to the case company for approval. In total, 11 stakeholders within the case company were interviewed. In addition, these 11 stakeholders had to use other employees to answer follow-up questions.

### Organization of PROs in each country

Organization of PROs and their relationship with an EEE manufacturer/importer is an important part of the WEEE organization in Europe. Therefore, interviews with PROs were needed. For the PRO interviews, two strategies for selection were used. All subsidiaries were asked about contact information in their PROs. Some subsidiaries had a close connection to their PRO, but as mentioned in several studies, the EPR organized with PROs may distance companies from their EPR obligations (Corsini et al. 2017; Andersen et al. 2020). Several of the subsidiaries had a peripheral relationship with their PROs, and many did not have a specific dedicated contact person. In these cases, the PROs were contacted at their official email addresses and by phone. Some PROs were difficult to contact, and some directly refused to participate in this study. The WEEE forum is an association of several WEEE PROs all over the world. Via this network, the author was able to contact more PROs to obtain enough data for the study.

To support the interview, an interview guide was developed. The interviews consisted of 14 questions addressed to the stakeholders from the different subsidiaries and 16 questions to the PROs. Both sets of questions were grouped into five topics listed in Table 3. The topics were the same for both the PROs and subsidiaries, but the questions were slightly different. The topics were linked to the supporting research questions mentioned in Section 1.

**Table 3:** Topics covered by the interview

Number	Topic
1	E-waste handling related to the WEEE regime WEEE in general, PRO organization, fee collection, and reporting. Quality of work done related to reporting
2	Changes in the WEEE directive Are the units aware of the changes? Consequences
3	Increased circularity in WEEE About WEEE changes and if they have improved circularity in the loop. Other improvements
4	WEEE administration and management How easy/hard is it to fulfill the WEEE obligations?
5	PRO collaboration About selected PRO, relationship, improvements, and auditing

All the subsidiaries and the PROs were unique legal entities. In total, 17 stakeholders were included in the interviews, and these stakeholders represented 12 legal entities in eight European countries. Table 4 summarizes the different interviews, including interview type and duration. Follow-up questions by email or phone are not included in the table.

**Table 4:** The 12 interviews

Interview number	Country/e-actor	Stakeholder	Type	Duration
1	Norway/e-manufacturer subsidiary (E1)	1. Accounting manager 2. WEEE reporting responsible 3. QM manager 4. Manager PA 5. Manager PD	In-person In-person In-person Phone Phone	2.5 h 0.5 h 0.5 h
2	Norway/PRO (P1)	6. Market and communication manager	Phone	1 h
3	Denmark/e-manufacturer subsidiary (E2)	7. Market manager	Phone	1 h
4	Germany/e-manufacturer subsidiary (E3)	8. Logistic manager	Phone	1 h
5	UK/e-manufacturer subsidiary (E4)	9. Product manager	Phone	1 h
6	UK PRO (P2)	10. External affairs manager	Phone	1 h
7	Ireland/e-manufacturer subsidiary (E5)	11. Financial controller	Phone	1 h
8	Ireland/PRO (P3)	12. Compliance and membership manager	Phone	1 h
9	The Netherlands/e-manufacturer subsidiary (E6)	13. Financial controller	Phone	1 h
10	Estonia/e-manufacturer subsidiary (E7)	14. Financial manager	Phone	1 h
11	Estonia/PRO (P4)	15. General manager 16. Management assistant	Phone Phone	0.5 h 0.5 h
12	Belgium/PRO (P5)	17. Spokesperson	Phone	1 h

**Fig. 4:**
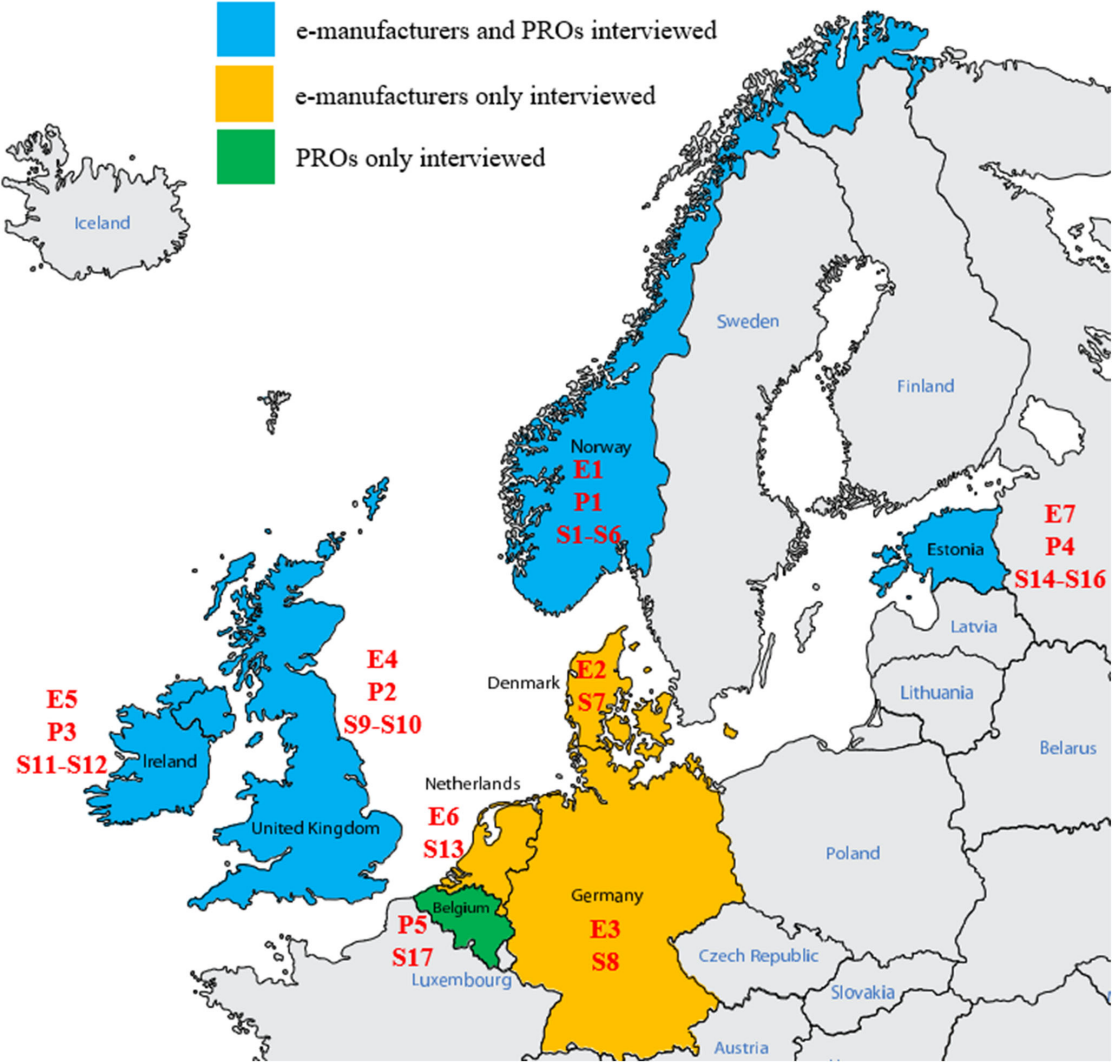
Countries and interview types. E: e-manufacturer, P: PRO, S: stakeholder

The country-wise interviews are categorized into three approaches: countries for which both e-manufacturers and PROs were interviewed, countries for which only e-manufacturers were interviewed, and countries for which only PROs were interviewed. This division is illustrated in Fig. 4, which identifies the seven interviewed e-manufacturers (E1–E7), the five PROs (P1–P5), and the 17 stakeholders (S1–S17).

Content analysis was used to derive meaning from the interviews. Content analysis allows one to make inferences by identifying certain characteristics of the data (Bryman 2012). This wide interview approach was necessary to gain different perspectives on the research questions. The interview results were analyzed with data from business documents, allowing for triangulation (Bryman 2012) in the analysis. Access to the resources needed for the successful completion of this research was directly granted because the author had, until recently, worked at the company. The case study provided in-depth knowledge of the company’s handling of the WEEE directive since this study investigates how one manufacturing EEE company treated its WEEE obligations. According to Yin (2018), a case study is suitable for this kind of research.

## The case company, its subsidiaries, and its PROs

The case company is a multinational EEE manufacturer that develops, produces, and distributes EE equipment in a professional (business-to-business) market. Both production and distribution are international, with factories, distribution, and sales units all over the world. The head office is located in Norway, and the company has several thousand employees. The case company has a significant market share for its product portfolio in the Nordics, in Europe, and for some product ranges and customer segments worldwide. The European part of the operation has been covered by the WEEE legislation since it was introduced (Union 2002). Presently, the WEEE legislation of 2012, with amendments (Union 2012), regulates the company’s EPR related to WEEE. For each EU/EEA (European Economic Area) country in which the case company is present, the company needs to be a member of an authorized PRO. These PROs may differ from country to country depending on how they are organized and financed, as well as other characteristics. The PROs may be non-profit member organizations, but they may also be commercial companies. Financing PROs’ operations is achieved by collecting an environmental fee when the EE equipment is sold. The environmental fee may differ from one EU/EEA country to another, depending on both the size of the environmental fee and on how this fee is calculated. The environmental fee may be calculated based on the value, weight, or the volume/size of the product; the material the product is made of; or a combination. In some countries, it is expected to separate the environmental fee on invoices and other business documents, while in other countries, this distinction is not necessary. Due to the different WEEE regimes, the case company must keep track of several WEEE dimensions when it places EE equipment on the European market. The company’s environmental commitment is stated in its environmental policy and in its values. Several of the company’s production units are ISO 14001 environmental management systems certified (ISO 2015).

The WEEE legislation is a European directive. Each member state in the EU/EEA is responsible for implementing the WEEE directive into its national laws. This implementation may differ from country to country and is related to several dimensions, such as collective or competitive systems and financing models. To identify similarities and differences in how the case company organized its EPR related to WEEE, several European parts of its operation were addressed in the interviews. Since the company’s head office is in Norway, many common functions are located there. Some of the answers were valid in all countries, but some were country specific, where stakeholders in one country only focused on WEEE issues related to their country. As an overall policy within the case company, each country unit is responsible for fulfilling the national WEEE regime. The case company has manufacturing units in several European countries. The manufacturing units serve their own country, as well as other countries via export. Sales in all European countries in which the case company is present are organized into sales units for each country. Although the sales units and production units in one country may be the same legal entity, the internal organization is divided into sales and production. All sales units are therefore represented as importers of EEE into their country, but only the countries with production units in their own county are represented as manufacturers of EEE in that specific country.

### Producer responsibility organizations (PROs)

Organization of PROs is an important part of the implementation of the WEEE directive. Since this organization is handled differently in Europe, and since there are variations in the interaction between the case company’s subsidiaries and PROs, interviews with some European PROs were conducted. Some of the PROs were selected from the case company; some were not. In total, five PROs, in Norway, the UK, Estonia, Ireland, and Belgium, were interviewed.

The PROs in Norway, Estonia, and Ireland were connected to the case company while those from Belgium and the UK were identified via the global PRO network WEEE forum. Most of the interviewed PROs had their own resources handling external request like this case study, typically managers within communication, marketing, or compliance departments. The PRO in Estonia did not have employees with this function. For Estonia, the head of the PRO answered some questions, and his assistant answers others. Belgium was selected although no subsidiaries were interviewed from that country due to their special organization. Belgium was also selected because the author had to exclude one PRO interview from a nearby country as the interview process was stopped before it was completed due to a lack of time and resources. Although some data were collected from this PRO, it was skipped because the interview was not completed. Table 5 presents some of the characteristics of the PRO organization in the actual countries. Information about the characteristics was obtained from the interviews, partly from the PROs and partly from the e-manufacturers.

**Table 5:** Organization of PROs in each country

Country	Organization of PRO
Norway	Competing collective system. 5 PROs. Commercial and non-profit
Denmark	Competing collective system. 5 PROs. Commercial and non-profit
UK	Competing collective system. 28 PROs. Commercial (27) and non-profit
Ireland	Competing collective system. 2 PROs. Commercial and non-profit
The Netherlands	Competing collective system. 4 PROs
Estonia	Competing collective system. At least 2 PROs
Belgium	Collective system. One PRO
Germany	N/A (see Section 5.1)

## Results

In this section, the results of the interviews are presented. These results are grouped according to the five supporting research questions listed in Section 1, which are linked to the topics covered by the interviews listed in Table 3.

### RQ1: How does a national e-actor view its e-waste handling related to the WEEE regime?

Regarding the first question of how a manufacturer views its e-waste handling in relation to the WEEE regime, all stakeholders perceived WEEE as a positive environmental contribution. All stakeholders also mentioned that the WEEE treatment was a well-functioning arrangement and that, as an EEE manufacturer, it was fair they took responsibility for financing and arranging collection and EoL treatment of the products they produced. Although it was not mentioned in the questionnaires, none of the subsidiaries focused on free riders or any negative competition conditions that may result from the WEEE.

The collection of environmental fees was implemented differently in the different countries. The easiest/lowest burden put on EEE manufacturers or importers was in Norway. Although Norway is not a member of the EU, membership in the EEA means Norway must implement the WEEE legislation. The Norwegian part of the case company, the Norwegian PRO, and several European subsidiaries stated the Norwegian model for calculating fees was an easy and elegant way to collect environmental fees. The fees are a percentage of the sales price for certain product categories. The calculation is based on the same concept as the calculation of value added tax (VAT). Although it is not decided by law, the market expects the calculated fee to be visible on business documents. This requirement is easy to implement since the VAT calculation concept can be used. The current PRO stated that figures on imported EEE were not reported by the importers. These figures were extracted from custom data from Directorate of Norwegian Customs. From these data, the PRO could extract data about the importer and data about the products, such as categories and weights. As far as the PRO knew, Norway was the only country using this approach. In contrast, Denmark’s approach was seen as the most difficult. In Denmark, there were often changes in how the environmental fee was calculated, and there was a significant cost with these changes. In some countries, the environmental fee is expected to be visible on business documents, such as invoices and order confirmations. None of the PROs stated that this requirement was regulated by law, but it was expected within some industries. For the case company, they displayed the fee on invoices in Norway, Denmark, and Ireland. Except for in Norway, this requirement was considered an extra burden, increasing the complexity. In Ireland, the interviewees stated that the fee collection was easy, despite there being different categories and the fee presented on invoices. However, that difference was due to someone configuring an internal WEEE IT tool, which handled everything related to WEEE fee calculation and reporting (see Section 5.5.1). In Estonia and UK, the case company only collected the environmental fee on an overall basis, which was straightforward. In some countries, like Denmark and Ireland, there is a direct connection between collection of the environmental fee and reporting of EEE placed on the market. When these countries collect environmental fees, they have a direct link to the kind of EEE they put on the market. In these cases, fee collection and WEEE reporting are based on the same principles. Here, WEEE reporting is a result of all environmental fees collected in a specific period. When there is no visible environmental fee, the manufacturer or importer has to cover these costs as a part of its cost calculation. In these cases, the number of EEE placed on the market, including categories, is the key for the manufacturer/importers environmental fee paid to the PROs. If the product categories fit the case company’s product portfolio in a present country (i.e., all products fit into one category), reporting is easy. This was the case in UK and partly in Estonia. In the Netherlands, the categories fitting the case company were more divided than described in the WEEE directive, which made reporting challenging and demanding. However, since this calculation could be completed with accumulated data, the process was easier than if calculations had to be done up front, where it is necessary to display the environmental fee on business documents.

Respondents from the two countries reporting that their WEEE reporting and fee calculation was challenging, Denmark and Netherlands, argued to harmonize implementation of the WEEE between EU countries. Several PROs also addressed this argument. One member of the WEEE forum explained that this goal also was on their agenda. As she said, most of the actors placing EEE on the European market are international players all over the world. She also stated that, although many of the EEE manufacturers are large companies, the expertise in each country seems limited. WEEE is only a small part of an EEE manufacturer’s responsibility, normally followed as a small part of one person’s work tasks. Even if the manufacturer wants to accurately fulfill their WEEE obligations, resources and knowledge are limited. The Irish PRO supported this point of view and explained that educating manufacturers and importers about WEEE was a time-consuming and challenging task. It may take years for an EEE importer or manufacturer to understand its WEEE obligations, and normally, only one person in the company has this knowledge.

Responses from the German part of the company were different from the other responders. Due to some changes in their products mix, German interviewees no longer reported or collected any fees. This change was seen as positive; the subsidiary was released from some “administrative burdens.”

### RQ2: How has a national e-actor reacted to changes in the WEEE directive?

For the second research question, the focus was on the categorization changes in the directive (from 10 to 6 categories) and on the changes in the different targets. The interviewer asked whether the respondents were aware of the changes and what impact the changes had caused. From the manufacturer/importer side, five of the seven responders were unaware of both the changes and any resulting impact. Several of the responders stated that they did not have to focus on WEEE changes because the PRO handled it for them. Two responders were aware of the category changes because these changes had resulted in changes in their reporting to their PROs. These respondents were the same ones who stated that WEEE reporting and fee calculation were challenging and argued for a more harmonized WEEE implementation in Europe (see Section 5.1).

All of the PROs were familiar with the WEEE changes. Regarding to the changes in categories, some countries have decided to have different categories than the WEEE directive. In the UK, they have 14 and in Norway 8. Some of these changes are only related to categorization, but some also expand the scope of WEEE treatment. The changes in targets were known by all the interviewed PROs.

### RQ3: How does a national e-actor respond to the increased circularity focus in the WEEE directive?

As a follow-up to an earlier study conducted by the same author (Andersen et al. 2020), the participants were asked about circularity and WEEE. In the previous study, there were indications that there is a mismatch between the ambitions in the WEEE directive and a company’s approach related to circularity in the EoL phase of an EEE product. None of the seven EEE manufacturers/importers were aware of the higher targets in the directive nor of the change of target from “recycled” to “prepared for re-use or recycled.” None of the importers/manufacturers of EEE could connect any changes in the WEEE directive to a more circular approach related to their products at all. They had observed an increased focus on this issue, but more on a general level. In particular, the subsidiaries who only imported EEE stated that this was outside their control.

For the PROs, the answer was different. All of them agreed that changes in the directive were ambitions for higher focus on circularity within EEE manufacturer’s development of products. However, none of the PROs had figures/reports proving this assumption. As one PRO stated, “the reuse of EEE is about 1%. If we want to increase this, there should be own targets on reuse of EEE” (translated to English by author). The same respondent also explained that there are re-use initiatives, like second-hand online marketplaces, but these initiatives are outside the WEEE regime since WEEE only acts when someone tries to handle EEE as waste. As another PRO stated, recycling and re-use are important for CE, but WEEE can conflict with CE. For example, there are no incentives for long-lived products. The manufacturers or importers pay the same fee whether a specific product lives for 1 year or 30 years. One responder mentioned eco-modulation (discounts or fees related to the products’ sustainability) and servitization as more important for CE than WEEE.

The PROs were also asked about product information from EEE manufacturers to WEEE collecting and recycling factories. The collector or recycler of WEEE should have easy access to information about the products from the manufacturer. As the WEEE processes are today, none of the PROs was aware of this sharing of information being regularly used. Typically, if there was a boost in WEEE from a specific EEE category, the recycling plant had to build competence in these products. One example mentioned was electricity meters used to measures the amount of electric energy consumed for billing purposes. In one country, manual meters had recently been replaced with meters that automatically communicate the usage of electricity to electricity retailers. The recycling plant was not familiar with this kind of equipment, and the PROs had to map the content of the different equipment. Mapping was completed in collaboration with the manufacturers of the meters.

Two of the PROs stated that the recycling and recovery process in the recycling plants was industrialized, for example, with disassembly lines. Everything not supporting process automation was considered irrelevant. The other PROs were more positive about this kind of collaboration. As one PRO stated, “we are the one who can link manufacturers to recyclers.” The PRO working as a monopolist also responded that establishing this product information flow was probably easier in their country because their organization was the only PRO.

One question asked whether marking products with unique product identifiers (i.e., QR codes) to identify products and their content could be helpful. Several PROs did not see this practice as an improvement because it would slow operation in the recycling plants. One PRO had tried to initiate a research project using RFID (radio-frequency identification) to identify products but was informed that the RFID would need electricity on the equipment to identify the products. The project was therefore cancelled. Another PRO was considering artificial intelligence to improve sampling before recycling. The PROs focusing on recycle plants as an industry did not see unique product identifiers as a valid improvement, but one PRO had a specific exception. The manufacturing date of a product identifies whether hazardous materials is in use. The PROs pragmatic about the information flow were also more positive about the usage of unique product identifiers. As one said, “we need to have a better idea of the waste stream,” but she also stated that information sharing had to be done on an international, or at least European, level. Another PRO stated that digitalization of only the return process would not benefit the WEEE treatment; in that case, the complete process, including bringing EEE to the market, had to be digitalized. This process would also benefit knowledge about the waste flow of EEE outside the WEEE directive.

### RQ4: How is the WEEE directive managed from a national e-actor’s perspective?

All the subsidiaries had an overall positive opinion about the WEEE directive. However, in several of the countries, there was a fear of making mistakes. To be labeled as a “free-rider” or a company that did not have control of its environmental obligations was seen as devastating. As two of the PROs stated, because members may be afraid of being prosecuted, they may report products outside the WEEE scope as WEEE products to ensure they do not underreport. Both the importers/manufacturers and the PROs were asked about the challenges fulfilling the WEEE directive related to reporting and fee calculation. On a 6-point scale (‘very easy’, ‘easy’, ‘moderate’, ‘somewhat hard’, ‘hard’, ‘very hard’), the answers were between ‘very easy’ and ‘somewhat hard’, with an average between ‘easy’ and ‘moderate’. The PROs’ answers tended to be a bit more toward ‘moderate’.

The interview also mapped the different branch offices and identified how close their relationships were with their PROs (see Section 5.5.2). There was a correlation between how confident each country was about fulfillment of its WEEE obligations and how close their relationship was with its PRO.

Although some of the countries reported challenges related to fee collection or reporting, all respondents, including the PROs, indicated that the administration costs related to collecting environmental fees and reporting EEE placed on the market were low.

### RQ5: How do national variations in the WEEE directive implementation affect e-actors?

#### E-manufacturer subsidiaries

Findings from the interviews listed in Sections 5.1 through 5.4 document both similarities and differences between countries regarding how the case company has been affected by the WEEE directive. The overall similarity is the positive perception of their EPR related to WEEE. All respondents stated that their WEEE obligations are fair. Furthermore, respondents commonly explained they focused on WEEE as waste, missing some of the circularity intention. There are clear indications that knowledge about the WEEE and circularity in WEEE is not built in the e-manufacturer organizations but in the PROs.

However, the interviews also identified differences. Based on the findings, this study has determined the following. Environmental fee calculation and reporting are completed in significantly different ways in the different subsidiaries. Visibility of the environmental fee is different, from a common visible fee across the supply chain to a common not visible fee to an internal cost component only communicated between the subsidiary and the PRO. There are also differences related to how much effort the subsidiaries use to fulfill their WEEE obligations and regarding each subsidiary’s confidence in following the WEEE directive.

To fulfill the WEEE requirements, the case company has, over the last 15 years, developed an information and communication technology (ICT) tool to support its WEEE obligations. The tool was built based on data from other ICT systems, such as enterprise resource planning, product lifecycle management, and customer relationship management, to identify products, suppliers, and customers related to WEEE obligations. The tool is used not only to calculate environmental fees but also to report on EEE. The ICT tool is used in about half the EU/EEA countries where the case company is represented.

#### PRO collaboration

Most of the PROs connected to the case company are defined as non-profit organizations. Only in the UK are the connected PROs defined as commercial. All five interviewed PROs were non-profit organizations. Several of the PROs defined the companies they supported as members. In one country, the PRO wanted to be seen as an internal part of the business it supported. While many of the respondents from the case company were unsure about details within the WEEE directive and its national implementation, the PROs had clear answers. Some respondents from the subsidiaries answered questions with statements like, “we do not have to focus on these issues, our PRO is handling this for us.” Thus, knowledge about WEEE and how it is implemented in each country resides with the PROs not the case company.

Most of the interviewed PROs also provided producer compliance for batteries and two PROs also did packaging. The three EPR obligations, WEEE, batteries, and packaging, seem preferred as bundled waste handling compliance schemes both for PROs and for companies’ operation under these legislations.

During the interviews, manufacturers/importers had no critical or negative feedback about their PROs. The negative feedback that was provided mainly concerned the implementation of the regulation in national laws and suggested the national implementations should be harmonized in Europe or that the national implementation should be as easy as possible. Several importers/manufacturers had seen a decreased level of environmental fees financing the EoL treatment, which indicated the PROs were competitive and increasing their competitiveness by decreasing the EoL cost for the EEE.

Although all respondents from the subsidiaries had positive perceptions about their PROs, in some countries, distance between the PRO and the EEE manufacturer/importer was apparent. When the author interviewed the subsidiaries, he also wanted to contact their PROs. In some countries, this contact was easy: the manufacturer/importer had a contact person in the PRO, and arranging an interview was simple. In other countries, there was no formal contact, and the author had to contact the PRO via the PRO’s official channels (email and phone). None of the PROs responded to requests via email. Contacting the PROs via phone resulted in contact with two more PROs: one full interview and one limited (which was excluded from the results). There was a clear difference between the importers/manufacturers who had a key contact and those who did not.

At the end of the interviews, the interviewer asked about auditing the WEEE compliance. Two PROs stated that they did regular WEEE audits for their clients. One PRO said that the organization controlled the clients on a regular basis, but since this practice was not performed by auditors and was not considered a formal audit, she would not call it an audit. The last two PROs did not conduct organized audits, only ad hoc checks, especially if they suspected that something was wrong or to identify free riders. The PROs also indicated that the quality of their clients’ work related to WEEE compliance was high. While three of the seven EEE importers/manufacturers were unsure whether they correctly followed their WEEE compliance, all PROs had a more positive perception about how their clients performed in this matter.

### Major findings

The results of the interviews can be summarized in five major findings. First, there are huge deviations in how an e-manufacturer needs to report its activities related to products put on the market. Second, this deviation is also present in how an e-manufacturer pays for EoL treatment of its WEEE via environmental fees, from overall calculations to specific environmental fees based on product properties, such as product type or weight. Third, e-manufacturers are focused on following their EPR obligations. This focus is so strong that they may over-report their activities to ensure that they are fulfilling their responsibilities. Fourth, both e-manufacturers and PROs are satisfied with the organization of EoL treatment: the e-manufacturer finances the PROs to fulfill its EPR. However, this method of EPR organization may distance an e-manufacturer from its effort to make its products more circular. This distance may make the transformation to a more circular economy within EEE more difficult. Fifth, e-manufacturers see WEEE and the WEEE directive as a regulation to handle waste. The waste focus may be in conflict with international ambitions to have a higher circular focus within EEE.

## Discussion

In this case study, stakeholders from an EEE manufacturer in seven European countries were questioned about their relationships to the WEEE directive. In addition, five PROs in Europe were asked about the same topics. The questions were related to the five research questions: (1) how does a national e-actor view its e-waste handling related to the WEEE regime, (2) how has a national e-actor reacted to changes in the WEEE directive, (3) how does a national e-actor respond to the increased circularity focus in the WEEE directive, (4) how is the WEEE directive managed from a national e-actor’s perspective, and (5) how do national variations in the WEEE directive implementation affect e-actors? This chapter is organized based on these questions.

### RQ1: How does a national e-actor view its e-waste handling related to the WEEE regime?

There was unanimous positive feedback that WEEE is important. However, there are differences in how this is achieved in Europe. Regarding environmental fees, in some countries, the fee financing the EoL treatment of EEE is a transparent amount visible across the supply chain. In other countries, this fee is a hidden cost included in a product’s sales price. In one country, the e-manufacturer only pays a percentage of its EEE turnover (based on categories), while in other countries, the actors have to pay fixed amounts based on an EEE product’s characteristics (e.g., category, weight, size, content). For all manufacturers or importers of EEE, the key purpose of reporting EEE placed on a market is to have a basis for paying the PRO for EoL treatment. For the PROs, it is also the basis for reporting figures to national and European environmental authorities. One country (Norway) used data from customs to identify imported EEE. Among others, categories, the weights of the products, and the importers were identified based on data from this source. This approach can be used for identification of EEE imported to Europe, but within the EU, there is no customs, so this method is not usable there. However, trade within the EU is reported via its Intrastat statistics, so numbers are available, but compared to custom clearance completed in advance of importing goods, Intrastat reporting is performed after goods have crossed a country’s border. Whether all necessary reporting dimensions are available in Intrastat is also unknown.

The interviewed e-actors believed differences in financing and reporting EoL within Europe created a challenging task. Nelen et al. (2014) proposed the following four WEEE recycling indicators for sustainable materials management: (1) weight recovery of target materials, (2) recovery of scarce materials, (3) closure of material cycles, and (4) avoided environmental burdens. The last two indicators seem to be missing in the existing WEEE regime in Europe. Presently, it is possible to trace what EEE each manufacturer brings into the market but not where and when the products are taken out of the market. A manufacturer with short lifetimes for its products is not treated differently than a manufacturer producing the same type of EEE but with longer lifetimes.

EPR can either be organized individually or collectively. All of the implementations studied here were collective, indicating that all the manufacturers/importers within one country pay the same share to handle EoL treatment of their products (Ameli et al. 2019; Massarutto 2014). Several respondents, both from the importer/manufacturer side and from the PROs, suggested a more harmonized regime for collecting fees and reporting. The manufacturer is global, but legislation is national. This has also been addressed by others, among them Kunz et al. (2018).

### RQ2: How has a national e-actor reacted to changes in the WEEE directive?

For the second topic, there are several differences in the findings. While all the interviewed PROs were familiar with both target changes and category changes, these changes were unknown to the majority of the importers/manufacturers. The importers/manufacturers familiar with the changes to categories were those who had to do the most detailed reporting on EEE placed into the market. None of the importers/manufacturers were familiar with the changes (usually higher) in the recovery and recycle targets. The distance between the PROs and manufacturer was documented by Atasu and Subramanian (2012), among others. They argue that the PROs’ organization makes it less favorable for the manufacturers of EEE to design products for recovery (DfR). Zoeteman et al. (2010) argue for a more active role from e-manufacturers to achieve more sustainable solutions. They divide WEEE handling into four stages from “doing nothing” to “high level recovery” in which repair, refurnish, and remanufacturing are important elements. To move to this higher stage, direct involvement from the e-manufacture is crucial.

### RQ 3: How does a national e-actor respond to the increased circularity focus in the WEEE directive?

The third topic investigated whether the changes in the WEEE directive resulted in an increased focus on circularity among the responders. While the PROs, at least to an extent, saw extended targets and “prepare to re-use or recycle” introduced as CE initiatives, none of the subsidiaries had observed these changes. The minimal focus on circularity related to WEEE was also mentioned in another study. Lu et al. indicated that the WEEE legislation is limited in terms of how to evaluate the potential for preparation for reuse (Lu et al. 2018). Furthermore, there are other barriers to improve circularity in WEEE. Parajuly and Wenzel (2017) identified obstacles when they collected and investigated reuse and recycle potential of nearly 5 tons of WEEE. They identified several barriers for reuse, much of it related to the current WEEE treatment focus on WEEE as waste, not a potential value for reuse and recovery. One recommendation was to label products with their manufacturing year to identify the product’s lifetime and help make the reuse decision. Similar findings were identified by Jaeger and Upadhyay (2020), who found that policies focusing on recycling and waste have a low effect on CE.

Introducing new technology to identify products and their manufacturers is considered challenging in the return process. Some studies focusing on this challenge. For example, Wang and Wang (2019) argue for usage of new technology in WEEE recovery to support improved remanufacturing operations. Digital twins and other Industry 4.0 components seem to have the potential to improve WEEE. Conti and Orcioni (2019) propose using cloud systems and RFID to trace WEEE. They also suggest a database structure for this tracking. These changes would result in efficient management of the reuse, repair, and recycle phase of products and components. Hayashi et al. (2019) used scanning of product labels to identify manufacturer and model names on discarded digital cameras. They used both the manufacturer’s logo and optical character recognition processing and were able to identify manufacturers and models even when the equipment was moving on a conveyor belt, a typical situation in a recycle plant.

### RQ4: How is the WEEE directive managed from a national e-actor’s perspective?

Administration of the WEEE directive, meaning collecting environmental fees, labeling products, and reporting EEE placed on the market, may be a challenging aspect of WEEE handling. How this administration is accomplished differs considerable across Europe. Both manufacturers/importers of EEE and the PROs were clear that it was not always easy to fulfill these obligations. Several PROs indicated that manufacturers/importers likely over-reported EEE to be “on the safe side.” A 2011 study about the former WEEE directive identified 72 reports an European EEE manufacturer had to deliver every year (Khetriwal et al. 2011). Although they argued for different systems, they also stated the need to establish common standards and harmonization of national and international legislation. Similarly, Bø and Baxter (2020) argue for more transparency within the reverse supply chain, but their focus is between PROs and transport carriers handling e-waste.

### RQ5: How do national variations in the WEEE directive implementation affect e-actors?

#### E-manufacturer subsidiaries

The European WEEE legislation allowing different implementations in European countries can be seen as both a strength and a weakness of the regime. Khetriwal et al. (2011) studied the implementation of the first WEEE directive in Europe. Although they argued for international harmonization of legislations, the differences they found in Europe gave insight into and knowledge about different ways to organize WEEE management. The differences in reporting in e-waste create challenges all over the world. Based on these challenges, the UN has devolved global guidelines for reporting and measuring WEEE (Forti et al. 2018). Europe is considered the best in class for WEEE treatment (Forti et al. 2020). However, within Europe, there are also deviations. Sousa et al. (2018) investigated the efficiency of the WEEE systems. They identified lack of available data related to EEE put on the market and environmental fee levels over Europe. Corsini et al. (2017) identified large variations in WEEE organization in Europe. They argue for harmonization in the implementation of WEEE in national legislation and for the organization of WEEE within Europe. The difference in if the environmental fee is visible along the reverse supply chain is not just present in Europe. There are variations all over the world (Ongondo et al. 2011). In a recent study from Canada, the e-manufacturers interviewed clearly stated a visible fee was the preferred option (Leclerc and Badami 2020). This visibility was seen as a way to communicate the e-manufacturer’s EPR commitment.

#### PRO collaboration

Organizing a company’s EPR via a PRO is a common way to fulfill a company’s EPR obligations. This study provides examples of both single and multiple organizations of PRO. In some countries, there are several PROs (from 2 to 28); in others, only one. Some PROs are organized as non-profit organizations, while others are commercial actors. The different organizations are well documented (Dieste et al. 2017; Forti et al. 2018, 2020; Leclerc and Badami 2020). There is also strong evidence that a competitive system of PROs provides economic benefits (Toyasaki et al. 2011). Others, stating “superior DfR incentives,” have argued that organizing EPR as individual producer responsibility results in a better connection between a manufacturer and the manufacturer’s focus on its products’ EoL phase (Atasu and Subramanian 2012). They also observe that a collective system may result in more free riding related to DfR.

## Conclusions, implications, limitations, and directions for future research

E-waste handling according to the WEEE directive is a long running and well-functioning waste management arrangement within Europe. Nordic countries and Switzerland are seen as frontrunners (Ylä-Mella et al. 2014; Forti et al. 2020) within Europe and on a global level. Recovery and recycle rates are high and increasing. In this study, 12 e-actors were interviewed about their WEEE obligations: manufacturers, importers, and PROs. Participants in this study, both from the industry side and from the PRO side, argued for more harmonization on WEEE legislations and implementation within Europe and internationally. The industry wants to fulfill its environmental obligations efficiently. The time and knowledge within WEEE in the industry is limited. These limitations should be used to fulfill the overall goals in EEE EoL treatment, not national specialties within the area. Although e-manufacturers are often large global players, much of the effort they use to meet their EoL obligations is expended on a national level. The EEE manufacturers and products are often global, but the EoL regime is local. The specific WEEE directive is in conflict with a CE idea because, in a CE approach, waste should be eliminated or minimized, while the WEEE directive only focuses on the lower levels within the waste hierarchy. Europe can coordinate its different directives and policies to harmonize the WEEE directive with other directives, like the eco-design directive (Union 2008a) and their CE policy (Union 2015).

Organizing EPR as collective systems is a success both from a manufacturer’s perspective and from the PROs’. A competing collective system was observed in at least six of the eight European countries studied. However, the collective method of organization for a company’s EPR distances the company from its EPR obligations. An individual system has better potential for this (i.e., related to DfR). Presently, no results that indicate a closed loop in the supply chain were found, knowing the amount of EEE a manufacturer brings into the market compared to EoL treatment of the same manufacturer’s WEEE. There are indications that e-manufacturers over-report their EEE contributions to secure fulfillment of their CSR. Technologies such as unique product identification systems and RFID may close the gap, but as the EoL process is organized today, there are many challenges to overcome before the loop can be closed. However, both products and supply chains are becoming more digital. To utilize emerging digital technology in the EoL treatment, the supply chain, including the EoL/reverse supply chain, should be seen as a whole. With this perspective, a combination of the collective (and competing) compliance schemes and individual EPR figures can be supported. However, to succeed, a long-time horizon is needed. Some products within EEE have lifetimes in the decades. If we do not focus on the EoL treatment in an early phase, an improved circularity cannot be fulfilled. Currently, WEEE treatment is handled as waste treatment, and e-manufacturers and partly the PROs are satisfied with the situation. There are also indications that organizing EPR with collective PRO systems may centralize knowledge about EoL treatment in the PROs, making e-manufacturers unsuitable to improve EoL treatment and circularity in their products.

This study has provided an understanding of practical problems in WEEE treatment from real-world cases. Organization of EoL treatment responsibility for PROs may decouple the producers from their EPR, making it difficult or impossible to close the loop in reversed logistics. This may also delay a transition to circularity in EEE products. Currently, the WEEE is focusing on waste, not circularity. E-waste handling might in the future not be coined WEEE, but CEEE, circular electric and electronic equipment to emphasize the shift. E-manufacturers are focusing on their CSR and EPR and are even willing to overperform to fulfill their obligations.

### Recommendations

Some actors perceive the implementations of reporting and fee calculation in the WEEE directive between countries in Europe as too complex. It is hard to determine why it has to be like this, especially when there is no correlation between this and how well the WEEE is handled in different European countries. It is also hard to argue for the significant variations in reporting and calculation of environmental fees between countries within Europe. This study calls for harmonization and simplification within this area to use maximum effort for environmental aspects, rather than administration. The organization of EoL using PROs has benefits and drawbacks. Both PROs and their clients see this as positive on environmental and administrative levels. However, it distances the manufacturers from their EoL obligations, especially related to circularity in their products. There is no link between the EEE an e-actor places on the market and the same e-actor’s amount of e-waste. Closing this gap is essential for a more circular approach within WEEE. Digital technology can support this goal.

Based on the research, the recommendations in four main areas are as follows:

1. Simplify WEEE reporting and financing.
2. Harmonize WEEE reporting and financing.
3. Strengthen governance of WEEE reverse supply chain to strengthen the manufacturer’s responsibility.
4. Focus on digital technology to link procurers of EEE to their WEEE obligations.

The first two recommendations should be addressed by policy-makers within Europe and on the international level. The third and fourth recommendations are valid on managerial and operational levels and may also have policy implications.

### Limitations and future research directions

This study is limited to one multinational EEE manufacturer, including study of branch offices in seven European countries and five PROs. The manufacturer only covers two WEEE categories. Although generalizing from a case study in certain scenarios might be possible (Yin 2018, 37–41; Flyvbjerg 2006), the present study has limitations regarding this aspect. Expanding the scope to companies handling other categories and including more countries in Europe may give more insight into how the WEEE regime performs in Europe. In the present study, the geographical focus was on the northern part of Europe. Focusing on the southern and eastern parts could provide interesting results. This study investigated the European WEEE directive. Other regions have organized their WEEE treatment differently. Comparing results from other regions can give broader insights into how e-manufacturers respond to their EPR in other regions or countries (e.g., the USA and China, which are also large generators of WEEE). Although there are some studies about WEEE and usage of emerging technology, more understanding of how technology can improve the EoL treatment of EEE seems to be of interest. In particular, how digitalization of the EEE supply chain can potentially affect the reverse supply chain to improve WEEE should be studied.

## Data Availability

Interview data, both recorded material and the transcribed results, are stored. Due to the agreement with NSD, data that can identify people will be anonymized or deleted latest 30 September 2023.

## Appendix

Categories including indicative list of EEE which falls within the categories of the old categories and non-exhaustive list of EEE which falls within the new categories

**Table:**

Categories before 15 August 2018	Categories after 15 August 2018
1 Large household appliances Large cooling appliances. Refrigerators. Freezers. Other large appliances used for refrigeration, conservation, and storage of food. Washing machines. Clothes dryers. Dish washing machines. Cookers. Electric stoves. Electric hot plates. Microwaves. Other large appliances used for cooking and other processing of food. Electric heating appliances. Electric radiators. Other large appliances for heating rooms, beds, seating furniture. Electric fans. Air conditioner appliances. Other fanning, exhaust ventilation and conditioning equipment. 2 Small household appliances Vacuum cleaners. Carpet sweepers. Other appliances for cleaning. Appliances used for sewing, knitting, weaving, and other processing for textiles. Irons and other appliances for ironing, mangling, and other care of clothing. Toasters. Fryers. Grinders, coffee machines, and equipment for opening or sealing containers or packages. Electric knives. Appliances for hair cutting, hair drying, tooth brushing, shaving, massage, and other body care appliances. Clocks, watches, and equipment for the purpose of measuring, indicating, or registering time. Scales 3 IT and telecommunications equipment Centralized data processing: Mainframes. Minicomputers. Printer units. Personal computing: Personal computers (CPU, mouse, screen and keyboard included). Laptop computers (CPU, mouse, screen and keyboard included). Notebook computers. Notepad computers. Printers. Copying equipment. Electrical and electronic typewriters. Pocket and desk calculators and other products and equipment for the collection, storage, processing, presentation or communication of information by electronic means. User terminals and systems. Facsimile machine (fax). Telex. Telephones. Pay telephones. Cordless telephones. Cellular telephones. Answering systems and other products or equipment of transmitting sound, images or other information by telecommunications. 4 Electronic and consumer equipment Radio sets. Television sets. Video cameras. Video recorders. Hi-fi recorders. Audio amplifiers. Musical instruments and other products or equipment for the purpose of recording or reproducing sound or images, including signals or other technologies for the distribution of sound and image than by telecommunications. Photovoltaic panels 5 Lighting equipment Luminaires for fluorescent lamps with the exception of luminaires in households. Straight fluorescent lamps. Compact fluorescent lamps. High intensity discharge lamps, including pressure sodium lamps and metal halide lamps. Low pressure sodium lamps. Other lighting or equipment for the purpose of spreading or controlling light with the exception of filament bulbs 6 Electrical and electronic tools Drills. Saws. Sewing machines. Equipment for turning, milling, sanding, grinding, sawing, cutting, shearing, drilling, making holes, punching, folding, bending or similar processing of wood, metal and other materials. Tools for riveting, nailing or screwing or removing rivets, nails, screws or similar uses. Tools for welding, soldering or similar use Equipment for spraying, spreading, dispersing or other treatment of liquid or gaseous substances by other means. Tools for mowing or other gardening activities 7 Toys, leisure and sports equipment Electric trains or car racing sets. Hand-held video game consoles. Video games. Computers for biking, diving, running, rowing, etc. Sports equipment with electric or electronic components. Coin slot machines 8 Medical devices Radiotherapy equipment. Cardiology equipment. Dialysis equipment. Pulmonary ventilators. Nuclear medicine equipment. Laboratory equipment for in vitro diagnosis. Analyzers. Freezers. Fertilization tests. Other appliances for detecting, preventing, monitoring, treating, alleviating illness, injury or disability. 9 Monitoring and control instruments Smoke detector. Heating regulators. Thermostats. Measuring, weighing or adjusting appliances for household or as laboratory equipment. Other monitoring and control instruments used in industrial installations (e.g., in control panels). 10 Automatic dispensers Automatic dispensers for hot drinks. Automatic dispensers for hot or cold bottles or cans. Automatic dispensers for solid products. Automatic dispensers for money. All appliances which deliver automatically all kinds of products.	1 Temperature exchange equipment Refrigerators, Freezers, Equipment which automatically delivers cold products, Air conditioning equipment, Dehumidifying equipment, Heat pumps, Radiators containing oil and other temperature exchange equipment using fluids other than water for the temperature exchange. 2 Screens, monitors, equipment with surface screens >100 cm^2^ Screens, Televisions, LCD photo frames, Monitors, Laptops, Notebooks. 3 Lamps Straight fluorescent lamps, Compact fluorescent lamps, Fluorescent lamps, High intensity discharge lamps—including pressure sodium lamps and metal halide lamps, Low-pressure sodium lamps, LED. 4 Large equipment (any external dimension more than 50 cm) including, but not limited to: Household appliances; IT and telecommunications equipment; consumer equipment; luminaires; equipment reproducing sound or images, musical equipment; electrical and electronic tools; toys, leisure and sports equipment; medical devices; monitoring and control instruments; automatic dispensers; equipment for the generation of electric currents. This category does not include equipment included in categories 1 to 3. Washing machines, Clothes dryers, Dish washing machines, Cookers, Electric stoves, Electric hot plates, Luminaires, Equipment reproducing sound or images, Musical equipment (excluding pipe organs installed in churches), Appliances for knitting and weaving, Large computer-mainframes, Large printing machines, Copying equipment, Large coin slot machines, Large medical devices, Large monitoring and control instruments, Large appliances which automatically deliver products and money, Photovoltaic panels. 5 Small equipment (no external dimension more than 50 cm) including, but not limited to: Household appliances; consumer equipment; luminaires; equipment reproducing sound or images, musical equipment; electrical and electronic tools; toys, leisure and sports equipment; medical devices; monitoring and control instruments; automatic dispensers; equipment for the generation of electric currents. This category does not include equipment included in categories 1 to 3 and 6. Vacuum cleaners, Carpet sweepers, Appliances for sewing, Luminaires, Microwaves, Ventilation equipment, Irons, Toasters, Electric knives, Electric kettles, Clocks and Watches, Electric shavers, Scales, Appliances for hair and body care, Calculators, Radio sets, Video cameras, Video recorders, Hi-fi equipment, Musical instruments, Equipment reproducing sound or images, Electrical and electronic toys, Sports equipment, Computers for biking, diving, running, rowing, etc., Smoke detectors, Heating regulators, Thermostats, Small Electrical and electronic tools, Small medical devices, Small Monitoring and control instruments, Small Appliances which automatically deliver products, Small equipment with integrated photovoltaic panels. 6 Small IT and telecommunication equipment (no external dimension more than 50 cm): Mobile phones, GPS, Pocket calculators, Routers, Personal computers, Printers, Telephones.
